# Supplementary material for: Establishment of Stable Immortalized Human Choroidal Melanocytes for Ocular Research
Source: Invest Ophthalmol Vis Sci. 2026 Sep 10;67(11):16. doi: 10.1167/iovs.67.11.16 (PMC13574216; doi:10.1167/iovs.67.11.16)
Supplement: Supplementary file 1 [file iovs-67-11-16_s001.docx]

**Supplementary data**

**Supplementary Table S1. Primers used to create the K4DT plasmids**

| Target | Sequence | Application |
| --- | --- | --- |
| CDK4-BamHI | CGCGGGATCCGCCACCATGGCTACCTCTCGATA | *Addition of BamHI/NheI restriction sites on to CDK4-R24C CDS |
| CDK4-NheI | GCTTCCGCTAGCCTCCGGATTACCTTCATCCTT |  |
| pLEFS-CDK4-R24C-tagGFP2-BleoR FWD | ACGTGCGGGACGTGGACATCGGTACCGGAAGCGGCGAGGG | Gibson cloning of CyclinD1 into pLEFS-CDK4R24C-tagGFP2-BleoR |
| pLEFS-CDK4-R24C-tagGFP2-BleoR REV | CACAGGAGCTGGTGTTCCATGGGACCGGGGTTTTCTTCCAC |  |
| pLV-CMV-cyclinD1- Puro FWD | TGGAAGAAAACCCCGGTCCCATGGAACACCAGCTCCTGTGC |  |
| pLV-CMV-cyclinD1- Puro REV | CCCTCGCCGCTTCCGGTACCGATGTCCACGTCCCGCACGT |  |

*Sequence amplified from pBABE-CyclinD1-CDK4R24C and cloned into pLEFS-akaLuc-tagGFP2-BleoR after BamHI and NheI.

**Supplementary Figure S1. Confirmed purity of NCM-K4DT lines at high passage.** Immunofluorescence staining showing positive expression for melanocyte markers SOX-10 (purple) and PMEL (green) in NCM-K4DT cells, while ARPE19 cells are negative. Only ARPE19 cells show positive expression for the RPE marker CRALBP (green). Nuclei are stained with DAPI (yellow). Scale bars: 100 µm.

**Supplementary Figure S2. Pilot evaluation of hTERT, Cyclin D1, and CDK4-Mut transgenes on NCM proliferative capacity.** (A) Schematic representation of lentiviral vectors used to express hTERT, Cyclin D1, or CDK4-Mut individually, as well as their combined expression (K4DT), in primary NCMs. (B) Short-term proliferation analysis showing population doublings of NCMs from four donors following transduction with individual transgenes (hTERT, Cyclin D1, or CDK4-Mut) or the combined K4DT construct. While individual transgenes did not consistently enhance proliferation relative to untransduced NCMs, combined K4DT expression resulted in increased population doublings across donors. Based on these pilot data, cultures expressing individual transgenes were discontinued after approximately three weeks. (C) Long-term growth curves comparing primary NCMs and K4DT-immortalized cells maintained in culture for up to 70 days. While the growth of primary NCM cultures plateaued, NCM-K4DT lines continued to undergo sustained population doublings over time.

**Supplementary Figure S3. Reduced proliferation of NCM-K4DT lines is associated with increased melanin accumulation.** (A) Phase-contrast images of NCM1-K4DT cells cultured in medium containing standard concentrations (100%) of cholera toxin (CT) and phorbol 12-myristate 13-acetate (PMA), reduced concentrations (50%), or no CT/PMA (0%). Over five days, cells cultured without CT and PMA were not viable, whereas cells maintained in 50% CT/PMA continued to proliferate, albeit more slowly than cells cultured under standard conditions. This trend was observed across all four NCM-K4DT donors (data not shown). Scale bars: 200 µm. (B) Quantification of population doublings for all NCM-K4DT lines after four days in culture under 100% or 50% CT/PMA conditions. Cells cultured in reduced CT/PMA exhibited a significant decrease in population doublings compared to standard conditions (*p* = 0.0179), consistent with a reduced proliferation rate. (C) Representative cell pellets from NCM-K4DT lines cultured for four days in medium containing 100% or 50% CT/PMA. Cells cultured under reduced growth-stimulatory conditions displayed visibly darker pellets, consistent with increased melanin accumulation. Scale bar: 5 mm.

**Supplementary Figure S4. NCM-K4DT lines successfully grew in low-serum and PMA/cholera toxin-free medium.** (A) Phase-contrast images of primary and K4DT cells from the NCM4 donor cultured in our standard melanocyte medium or in the commercial melanocyte proliferation medium CnT-40 during eight days. The proliferation was slower for cells grown in the CnT-40 medium, and there was a slight change in their morphology particularly evident as the cells reached confluence; cells cultured in the standard melanocyte medium displayed disorganized overlapping growth, while cells grown in CnT-40 had a tendency towards parallel alignment. This trend was observed across all four NCM-K4DT donors (data not shown). Scale bars: 100 µm. (B) Quantification of population doublings for all primary NCMs and NCM-K4DT lines after eight days in culture in standard or CnT-40 media. A two-way ANOVA at D8 revealed cells cultured in the CnT-40 medium exhibited a decrease in population doublings compared to standard conditions (main effect of medium type; *p* = 0.0048), consistent with a reduced proliferation rate. Post-hoc analysis revealed a significant difference between K4DT Standard vs. CnT-40 (*p* = 0.0186), but no significant difference between Primary Standard vs. CnT-40 (*p* = 0.1045). (C) Representative cell pellets from primary and K4DT cells from the NCM4 donor cultured for eight days in standard or CnT-40 media. Cells cultured in the low-serum and PMA/cholera toxin-free conditions displayed increased melanin accumulation. Scale bar:  5 mm.

**Supplementary Figure S5. Generation and validation of Luc2-tdTomato-expressing NCM-K4DT lines.** (A) Phase-contrast and fluorescence images demonstrating robust tdTomato expression across the four generated NCM-K4DT lines (left panel). Scale bars: 100 µm. Flow cytometric analysis confirming tdTomato expression in >93% of cells for all NCM-K4DT lines (right panel). (B) *In vitro* luciferase activity of Luc2-tdTomato-expressing NCM-K4DT lines prior to *in vivo* studies. Luminescence was measured across seven increasing cell numbers to generate standard curves, revealing a strong linear relationship between cell number and the luminescent signal, consistent with stable luciferase expression. (C) *Ex vivo* images of tumors and Matrigel^®^ plugs with fluorescence overlay. While strong tdTomato fluorescence was detected in tumors derived from the UM cell line 92.1, the fluorescence from NCM-K4DT Matrigel^®^ plugs was not detectable above background autofluorescence. Scale bars: 5 mm. (D) *Ex vivo* bioluminescence imaging of NCM2-K4DT Matrigel^®^ plugs performed immediately after euthanasia to assess cell viability and luciferase expression. Despite the absence of tumor formation, luminescent signal confirms the persistence of viable NCM2-K4DT cells 12 weeks post-injection. Due to variability in luciferin diffusion following intraperitoneal injection and the delay inherent to tissue dissection, luminescent signals could not be reliably captured at peak intensity; therefore, quantitative luminescent comparisons were not performed. Scale bars: 10 mm.
